# Intermetallic compounds in heterogeneous catalysis—a quickly developing field

**DOI:** 10.1088/1468-6996/15/3/034803

**Published:** 2014-06-11

**Authors:** Marc Armbrüster, Robert Schlögl, Yuri Grin

**Affiliations:** 1 Max-Planck-Institut für Chemische Physik fester Stoffe, Nöthnitzer Str. 40, 01187 Dresden, Germany; 2 Fritz Haber Institute of the Max Planck Society, Inorganic Chemistry, Faradayweg 4-6, 14195 Berlin, Germany

**Keywords:** intermetallic compound, complex metallic alloy, heterogeneous catalysis, methanol steam reforming, acetylene semi-hydrogenation, selective hydrogenation

## Abstract

The application of intermetallic compounds for understanding in heterogeneous catalysis developed in an excellent way during the last decade. This review provides an overview of concepts and developments revealing the potential of intermetallic compounds in fundamental as well as applied catalysis research. Intermetallic compounds may be considered as platform materials to address current and future catalytic challenges, e.g. in respect to the energy transition.

## Introduction

Intermetallic compounds, i.e. compounds comprising two or more elements located left and around the Zintl line in the periodic table [1], realize crystal structures which are completely or at least partly ordered and different from those of the constituent elements. The peculiar bonding situation in the compounds—caused by the unique combination of covalent and ionic interactions as well as the presence of conducting electrons—results in attractive combinations of crystallographic and electronic structures for potential applications in catalysis and surface chemistry. Beside their historical use as construction materials (cf bronze or brass), intermetallic compounds exhibit physical properties, such as superconductivity (MgB_2_ [2]), thermoelectricity (chlathrates [3]) or magnetism (SmCo_5_ [4]), making them interesting for fundamental research as well as for application. So far, the chemical properties of intermetallic compounds are only scarcely investigated, most research focusing on hydrogen storage capabilities [5] as well as corrosion resistance (e.g. FeSn_2_ [6])—one outcome with large industrial application is rechargeable nickel metal-hydride batteries based on LaNi_5_ [7].

Heterogeneous catalysis is a worthwhile target for intermetallic compounds. Catalysis accounts for an enormous added value worldwide and—besides its economic impact—enables feeding of the global population and reduced pollution of our environment. Further, it contributes significantly to the availability of functional and structural materials based on carbon such as polymers and will be one of the important pillars in a future sustainable energy infrastructure [8]. The latter comprises the use of sunlight (direct or indirect by wind- or waterpower) for the electro- or photochemically catalyzed water splitting as well as transforming the resulting hydrogen by catalytic processes into small molecules (e.g. methanol, ammonia or formic acid) for energy storage. The release-on-demand of hydrogen from these molecules to power hydrogen fuel cells or their direct use in fuel cells also requires appropriate catalysts.

Heterogeneous catalysis takes place on the surface of materials and involves in the simplest case only three general steps: adsorption of the reactants, reaction of the adsorbed reactants and desorption of the products. Thus, the adsorption properties of surfaces play a crucial role in heterogeneous catalysis. The factors determining the adsorption properties of surfaces can be grouped into two classes, i.e. electronic and geometric effects. Commonly, the first have a much stronger influence, while geometric effects can be used for ‘fine-tuning’ of the adsorption behavior [9]. In classical metal-based heterogeneous catalysis, transition elements as well as binary alloys are forming the materials basis. The reason for this restriction lies within the typically applied synthesis methods, e.g. impregnation of support materials, which aims at a high atom efficiency and large scale synthesis [10]. While achieving this combination of goals with a single metallic element can already be tedious, generating small and chemically homogenous particles of an alloy (formed by random substitution of one metal on the lattice of the second) is very challenging due to the complex chemistry and the many parameters during synthesis.

If successful, the resulting substitutional alloy reveals a modified electronic structure as compared to the monometallic elements causing different adsorption properties, which account for a desired change in catalytic behavior. Since crystal and electronic structure are dependent on each other, the mere exchange of atoms in a random way in a substitutional alloy—limiting the structural diversity to closed-packed arrangements—results in rather minor electronic changes. Another important aspect for the surface phenomenon of heterogeneous catalysis is segregation. Since the atoms in substitutional alloys have no strong site preference the atoms are rather mobile at elevated temperature and segregation becomes a common phenomenon under reaction conditions (see e.g. nickel and copper clustering in Cu_0.327_Ni_0.673_ [11]). By segregation, the adsorption properties of the alloy are lost, restoring the catalytic properties of the segregated element.

Besides substitutional alloys, many binary systems offer one or more structurally ordered intermetallic compound [12]. With their crystal structures being different from the usually closed-packed metallic elements, each intermetallic compound shows a specific electronic structure substantially different from the parent elements. The peculiar combination of their crystal and electronic structure results in unique adsorption—and thus catalytic—properties of intermetallic compounds as shown in this review. In contrast to substitutional alloys, a strong site preference is characteristic for many intermetallic compounds. Caused by the chemical bonding [13–15], the site preference can provide the stability, which is needed to exclude segregation and thus to maintain the crystal and electronic structure of the intermetallic compound under reaction conditions. This makes intermetallic compounds highly interesting materials to be studied in catalysis. Each of the more than 6000 binary intermetallic compounds known so far [16], has the potential to behave like a ‘new element’ in heterogeneous catalysis, opening a vast field to be explored.

In addition, intermetallic compounds can not only be used as such in catalysis, but can provide a unique precursor state resulting in catalytic materials, which are not accessible by other synthetic approaches. Besides the recent examples discussed below, the general idea dates back to Raney in 1925 [17]. Raney-type catalysts—representing leached intermetallic compounds and alloys—are today widely applied in the lab and in industry, e.g. the processing of vegetable oils into margarine [18] and in selective hydrogenation reactions. Despite their huge potential in heterogeneous catalysis, intermetallic compounds pose large hurdles concerning their successful synthesis as materials with high specific surface areas. Progress has been made in recent years, revealing that each intermetallic compound requires a specific synthesis protocol, but also that the compounds can be synthesized in an industrially feasible way.

The progress in the field of intermetallic compounds in heterogeneous catalysis is exemplified in the present review by two reactions, i.e. the semi-hydrogenation of acetylene and methanol steam reforming (MSR), where intermetallic compounds have made significant contributions in the recent years.

## Semi-hydrogenation of acetylene

**Figure 1:**
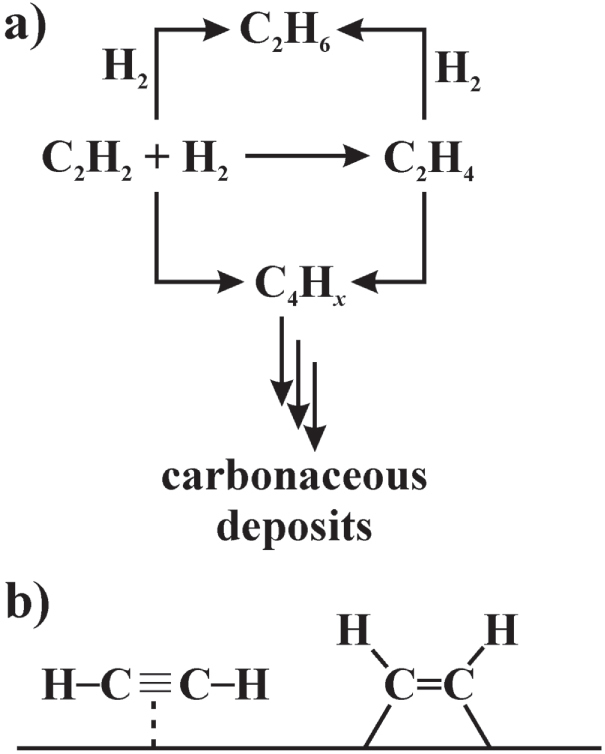
Reaction network of the semi-hydrogenation of acetylene (a); *π*- and di-*σ*-bonded acetylene (b).

Hydrogenation reactions are widespread in organic chemistry laboratories as well as in the chemical industry. While for total hydrogenation, e.g. transforming unsaturated C–C and C–*X* bonds to fully saturated hydrocarbons, several heterogeneous catalysts are used, partial and selective hydrogenation remains a challenging field. This applies to fine chemicals, e.g. partial hydrogenation of *α*, *β*-unsaturated aldehydes to the unsaturated alcohols [19], as well as to bulk petrochemicals. An interesting reaction of the latter class is the semi-hydrogenation of acetylene, which is used to remove traces of acetylene (around 1%) from the ethylene stream for the production of polyethylene (figure 1) [20, 21].

Polyethylene production is around 80 × 10^6^ tons per year [22], thus requiring highly active, stable and very selective catalysts to provide clean ethylene for the formation of polymers with controlled properties. The reason why acetylene has to be removed is that it acts as a poison for the ethylene polymerization catalyst, making it necessary to reduce its concentration to the low ppm regime [20].

This last requirement adds an additional burden to the catalytic material: the stronger adsorption of acetylene (than ethylene) and its subsequent preferential hydrogenation results in a rather high selectivity as long as the conversion of acetylene is low. Reducing the acetylene concentration to the low ppm regime requires nearly full conversion of acetylene, i.e. ethylene gains in coverage of the surface and is subsequently transformed to ethane without further economic value. Thus, the ideal catalyst for this reaction has not only to be very active and stable but should also possess an excellent selectivity in the conversion of acetylene to ethylene at nearly full acetylene conversion.

Based on the evaluation of numerous studies investigating the hydrogenation of acetylene and ethylene, Sinfelt, Sachtler and Ponec developed the active site isolation concept in the 1970s (see [23] and references therein). According to this concept, weakly adsorbed acetylene where the *π*-bonds are interacting with the surface (*π*-adsorbed acetylene, figure 1(b)) will be transformed to ethylene, while stronger di-*σ* adsorbed acetylene results in either full hydrogenation or the formation of carbonaceous deposits. The latter are undesired, since they lead to deactivation of the catalysts and reduce the time-on-stream. In industry, the beneficial site-isolation is since then realized by Pd-based alloys [24]. Here the palladium atoms provide the active hydrogenation sites, which are separated from each other by relatively inactive metals like silver or gold. In these substitutional Ag–Pd or Au–Pd alloys, the Pd atoms are randomly distributed in the crystal structure, thus not fully excluding the intimate contact of two or more Pd atoms. Resulting are geometrically large active sites allowing different adsorption configurations of the acetylene molecules. Thus the catalytic selectivity of these materials is intrinsically limited. This situation gets worse with time-on-stream, since palladium segregates to the surface. The increasing size of the active sites diminishes the selectivity, resulting in loss of ethylene as well as catalyst deactivation by carbonaceous deposits. To complicate things further, the Pd-based materials are often prone to sub-surface chemistry [25], especially hydride formation [26]. The activated hydridic hydrogen—the hydrogen–hydrogen bond is already broken—is highly reactive and does not distinguish between an unsaturated double and triple bond. As a result, ethylene selectivity is lost. In summary, the ideal catalyst possesses small and long-term stable isolated active sites. This allows for high selectivity for the semi-hydrogenation of acetylene and does prevent deactivation by carbonaceous deposits. In addition, the material is not prone to hydride formation to prevent total hydrogenation of ethylene and/or acetylene to ethane.

With the knowledge of these requirements as well as the drawbacks of the conventional catalysts, one can now start to think how to find materials fulfilling the profile and overcoming the obstacles. Intermetallic compounds are promising candidates, since—from the point of view of electronic transport—they fall into the category of metals (many of the intermetallic compounds show e.g. a metal-like temperature dependence of the electric conductivity) as the industrially applied alloys. This allows for a substantial density of states (DOS) near the Fermi level being prerequisite for facile activation of di-hydrogen as reactant. In addition, the huge structural variety allows for selection of compounds with isolated palladium atoms or small palladium ensembles, thus following the earlier proposed active site isolation concept. The latter feature already surpasses the structural ambiguity of substitutional alloys, since the crystal structure of such intermetallic compounds provides all palladium atoms within an ordered structure. In addition, the covalent bonding within the compounds leads to a much higher stability against segregation also under reaction conditions. This should lead to catalysts with excellent selectivity and long-term stability.

**Figure 2:**
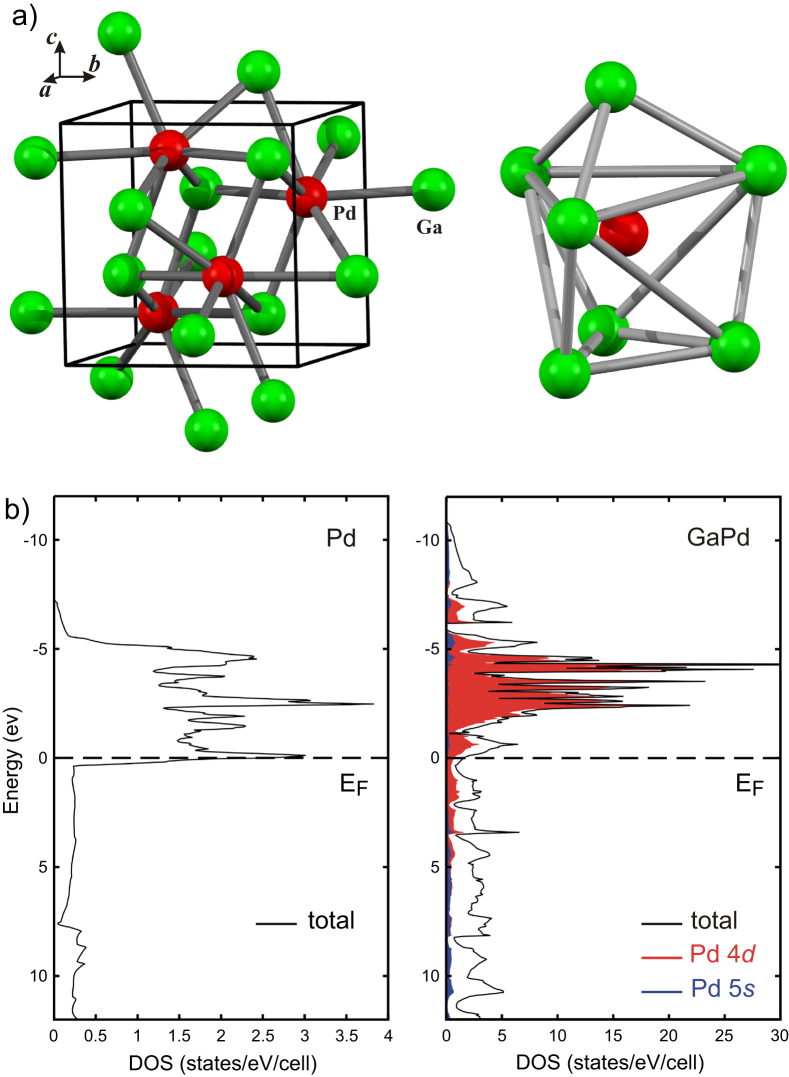
Unit cell of GaPd and coordination of palladium in GaPd (a). Density of states for elemental palladium as well as for GaPd (b).

The covalent bonding in the compounds leads to a strong alteration of the electronic structure. In figure 2 the electronic DOS of elemental palladium and of the intermetallic compound GaPd (FeSi type of crystal structure, space group *P*2_1_3, palladium atoms are only surrounded by gallium atom) are opposed [27].

The direct comparison reveals a strong shift of the Pd 4d states to lower energies, resulting in a higher degree of filling of the 4d states, thus a partial negative charge on palladium. The palladium d states in the intermetallic compound also reveal a smaller width than in elemental palladium—a direct cause of the isolation of the palladium atoms as well as their participation in the covalent interactions within the first coordination sphere. The resulting electronic structure for the Pd in GaPd resembles that of single palladium atoms in the gas phase, which would expose discrete d-energies. In addition, the DOS at the Fermi energy is strongly reduced. From the analysis of the electron density according to the quantum theory of atoms in molecules (QTAIM [28]), the charge transfer from Ga to Pd results in Ga^0.5+^Pd^0.5−^ [29].

Since the adsorption properties depend on the local surface electronic structure the changes of the bulk electronic structure shown in figure 2 may lead to a material which does not show catalytic activity at all because the adsorption of the reactants is either too weak or too strong. In the first case, the reactants will hardly be adsorbed on the surface and the reaction cannot take place. In the second case, the strong adsorption will lead to blocking of the active sites, leading to poisoning of the catalyst.

Hydride formation is detrimental for the selectivity in the partial hydrogenation of acetylene and has to be excluded under reaction conditions. Using an intermetallic compound as the catalyst does not automatically exclude formation of hydrides and the resulting changes [30]. In fact, the hydride formation ability of LaNi_5_ is exploited in Ni-metal hydride batteries [7]. Only a small number of intermetallic compounds have been investigated concerning their hydride formation behavior [5, 31]. Most hydride-forming intermetallic compounds have crystal structures based on close and closest packings and the hydride formation is mainly realized by interstitial hydrogen in tetrahedral and octahedral voids of the structures. As result, the compounds in question—even if they have crystal structures different to the close and closest packings—have to be investigated under reaction conditions.

**Figure 3:**
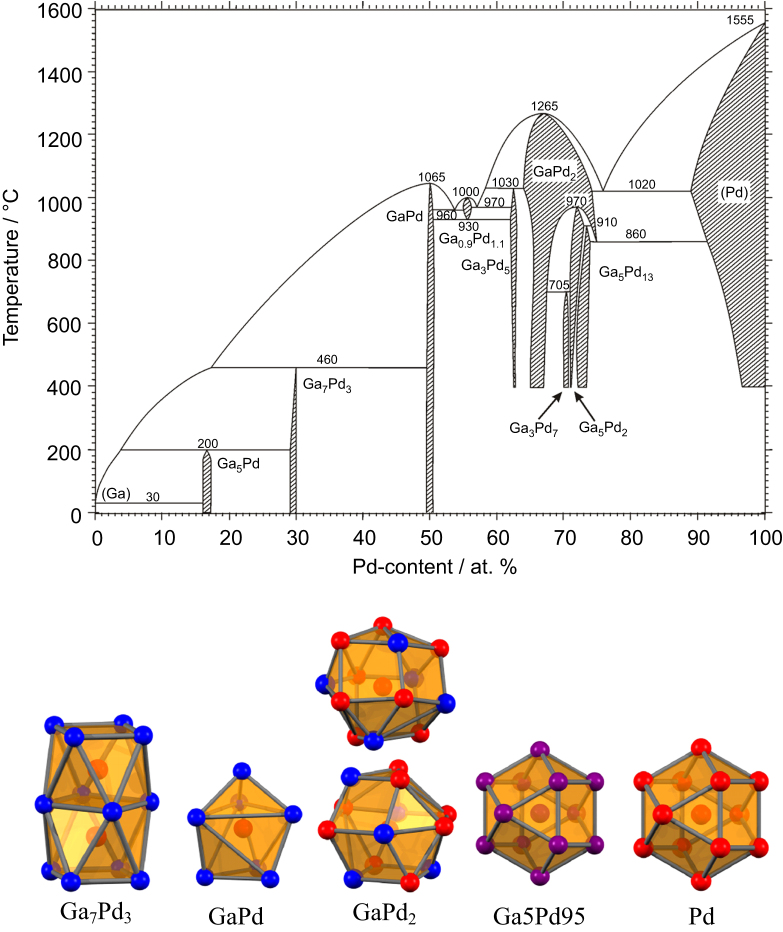
Ga–Pd phase diagram [32, 33] (top) and coordination of the palladium atoms in intermetallic Ga–Pd compounds as well as the alloy Ga5Pd95 and elemental palladium (bottom, gallium atoms are blue, palladium atoms are red; mixed occupancy in the alloy is indicated by purple).

Among the numerous possibilities in the large pool of intermetallic compounds, first investigations focused on the Ga–Pd phase diagram. On the one hand, the Ga–Pd compounds should exhibit a significant covalent bonding contribution. On the other hand, several compounds in this system realize crystal structures with significant site isolation of the palladium atoms (figure 3).

In elemental palladium each Pd atom is surrounded by twelve Pd atoms. This situation represents no isolation of the active sites. The size of the active sites is potentially only restricted by the geometric surface area. Adding small amounts of gallium introduces only minor changes: up to around 10 at. % Ga the closed-packed structure of palladium (Cu-type of crystal structure, space group *Fm* 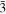 *m*) is maintained at 1020 °C—a substitutional alloy is formed. The random distribution of gallium in the alloy Ga5Pd95 results in an average number of 11.4 Pd atoms around each palladium. In addition, the distance between the palladium atoms does not change significantly. Increasing the Ga content, leads to the formation of GaPd_2_—an intermetallic compound which presents a fully ordered structure at the 1:2 composition (Co_2_Si type of crystal structure, space group *Pnma*, *a* = 5.4829(8) Å, *b* = 4.0560(4) Å, *c* = 7.7863(8) Å [34]). Here, palladium occupies two crystallographically non-equivalent sites. The prototype structure of Co_2_Si has a dual origin. It is described either as related to the face-centered cubic closest packing [35] or as derivative of the hexagonal structural motif of AlB_2_ [1]. As a result, the number of atoms in the first coordination sphere of Pd increases from 12 to 13 out of which only eight are palladium atoms. In addition, the closest Pd–Pd distance increases from 2.74 Å in the element to 2.8092 Å in GaPd_2_. Thus, the palladium atoms—even when being in the first coordination sphere of each other—are more isolated from each other than in elemental palladium. Further isolation of the palladium atoms is realized in the compound Ga_7_Pd_3_ (Ir_3_Ge_7_ type of crystal structure, space group *Im* 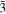 *m*, *a* = 8.7716 Å [36]). Here, all palladium atoms are located on one crystallographic site and each palladium atom is surrounded by a square antiprism of eight Ga atoms. The coordination is completed by one capping Pd atom, resulting in only one Pd–Pd contact (2.73 Å). Best Pd isolation is realized in the compound GaPd (FeSi type of crystal structure, space group *P*2_1_3, *a* = 4.8959 Å [37]), where the first coordination sphere of the palladium atom consists exclusively of seven gallium atoms. The closest Pd–Pd contact is with 3.00 Å around 10% longer than in elemental palladium.

**Figure 4:**
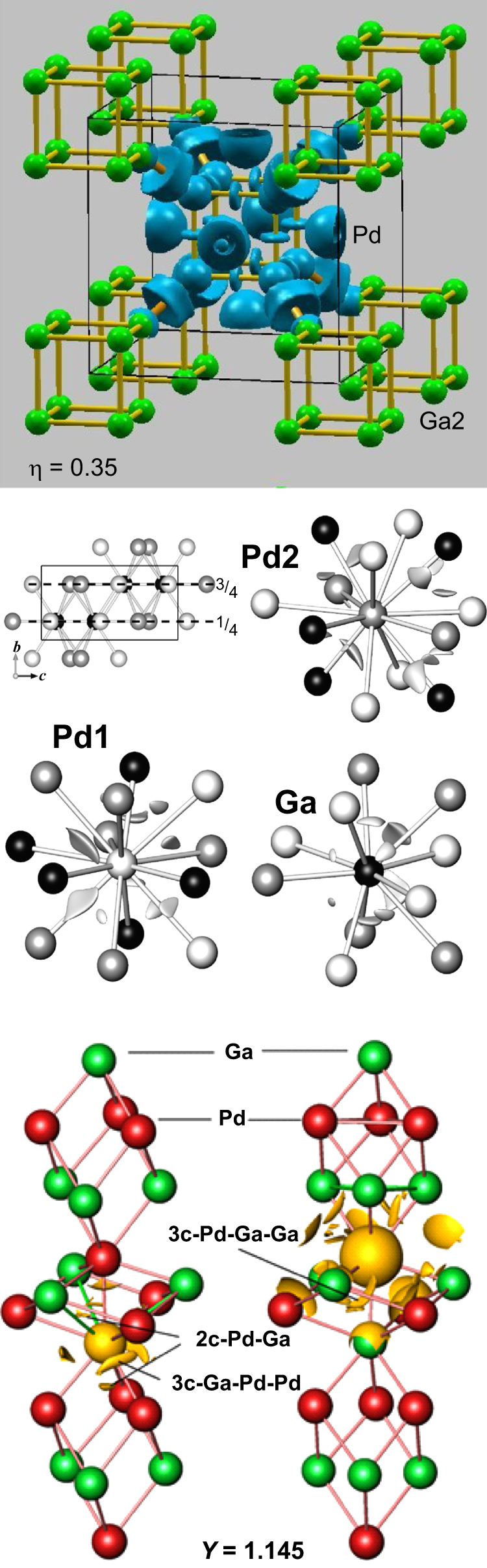
Electron localization function in Ga_7_Pd_3_, electron localizability indicator in GaPd and GaPd_2_ (from top) revealing covalent contributions to the chemical bonding in all compounds.

The covalent bonding in all these intermetallic Ga–Pd compounds has been investigated by means of quantum chemical calculations using the electron localizability approach. The spatial distribution of electron localizability indicator (ELI) yields basins representing atomic shells, lone pairs and chemical bonds in real space by describing the effect of local correlation of electronic motion [38]. In all Ga–Pd compounds, a three-dimensional network of covalent bonding is revealed by the calculations, thus embedding each atom in the compounds in a very specific chemical surrounding (figure 4) [27, 39, 40].

The specific bonding directly explains the low segregation tendency of palladium: to segregate to the surface, the atoms either would have to jump from one Pd position to a palladium vacancy or they would have to make use of Ga vacancies. While the first are rather far away from the palladium position, the second are so specific to gallium from a chemical bonding point of view that palladium atoms are not easily accommodated—both effects should result in high activation barriers for segregation. While this holds strictly for compounds holding 50 at. % palladium or less, the situation is slightly different in GaPd_2_, where the large number of Pd–Pd contacts allows for easier diffusion via the first path, thus making segregation more likely. As shown below, evidence for the expected, different behavior is experimentally observed.

**Figure 5:**
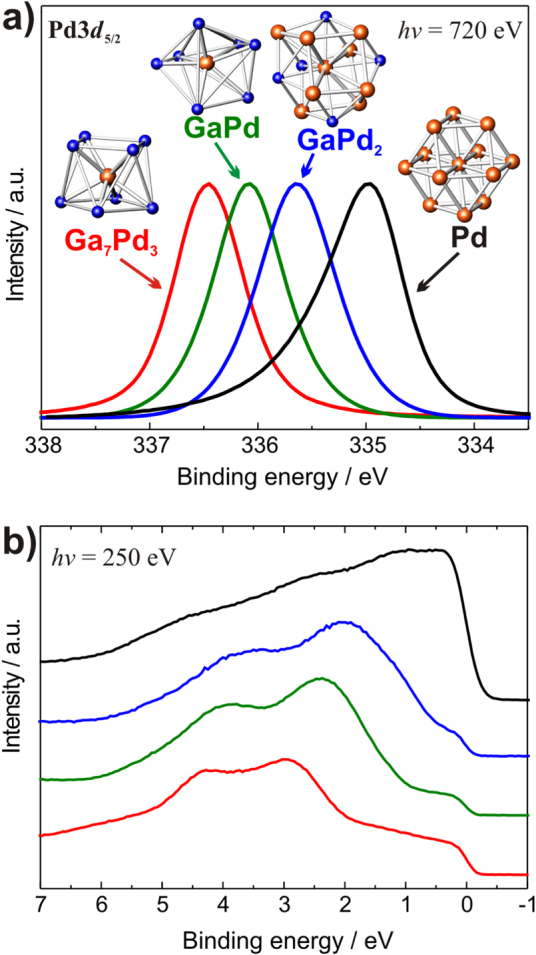
XPS spectra of the intermetallic compounds in comparison to elemental palladium: Pd3d_5/2_ core level spectra (a) and valence band spectra (b) (identical color code in both panels).

Before turning to the *in situ* behavior of the compounds, it is insightful to explore the electronic consequences of the compound formation. In figure 5 the experimental electronic structure of elemental palladium is measured by means of core-level as well as valance band x-ray photoelectron spectroscopy (XPS) and is compared to the electronic structures of the aforementioned intermetallic Ga–Pd compounds [41].

The higher the Ga:Pd ratio, the further the electronic states of palladium are filled, leading to a partial negative charge on the palladium atoms [27] following the expected charge transfer according to the electronegativity values (Pauling scale: Ga 1.81, Pd: 2.20). The more completely filled valence 4d-shell of the Pd atoms leads to a better shielding of the Pd 3d core hole as less relaxation through the valence band can occur: the photoemission becomes more atom-like. This and the filling of additional valence d-states push the core level to higher binding energy. This shift is thus no ‘chemical shift’ in the sense that if designates a positively charged Pd state at higher binding energy with respect to the reference bulk Pd. The expected shift is indeed observed experimentally by XPS studies, revealing an increase of up to nearly 2 eV of the palladium 3d states in comparison to elemental palladium.

The strong influences of the altered electronic structure on the adsorption properties of the compounds can be exemplified by temperature-programmed desorption spectroscopy (TDS) on single crystals. Taking carbon monoxide as a common test molecule for the surface of palladium-based catalysts, the (111) surface of elemental Pd and the GaPd:B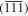 single-crystalline surface have been investigated using TDS [42]. CO desorbs only at around 510 K from Pd, but is already fully removed at 260 K from GaPd. This huge difference of 250 K is a direct consequence of the strong modification of electronic structure of the palladium atoms. To conclude, the covalent bonding and the different crystal structure of the intermetallic compounds lead to a higher degree of filling of the narrower d-states, resulting in a partial negative charge on palladium and strongly altered adsorption properties. This state of the materials, having a unique electronic and crystal structure in combination with electric conductivity, justifies the denomination of intermetallic compounds as ‘new elements’ in catalysis.

**Figure 6:**
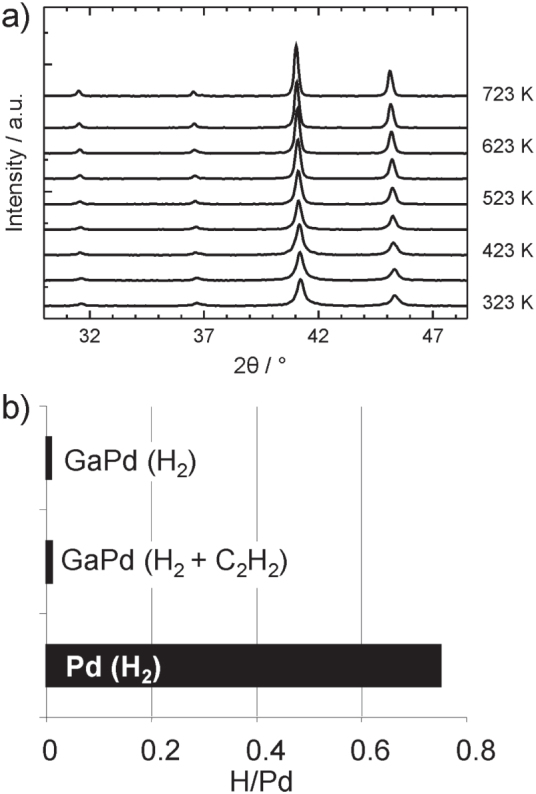
Temperature-dependent powder x-ray diffraction of GaPd in 50% H_2_ in helium (a). Results from PGAA for GaPd in pure hydrogen as well as under reactive conditions in comparison to elemental palladium (b).

To make use of these peculiar properties in catalysis requires that the intermetallic compounds are stable under reaction conditions—in the case of the semi-hydrogenation of acetylene with a special emphasis on hydride formation. The intermetallic compounds were investigated by *in situ* x-ray diffraction (XRD), extended x-ray absorption fine structure (EXAFS), prompt gamma activation analysis (PGAA), combined differential thermal analysis and thermogravimetry (DTA/TG) as well as near-ambient pressure XPS investigations to ensure bulk as well as surface stability in hydrogen-containing atmospheres and to be able to detect crystalline as well as amorphous phases in the bulk as well as in the near-surface region. Investigations by bulk sensitive methods like XRD, EXAFS and PGAA resulted in an excellent stability of the compounds. No hydrogen uptake, decomposition or phase transformation could be detected by XRD in atmospheres containing up to 50% hydrogen and temperatures up to 723 K [43] (figure 6).

**Figure 7:**
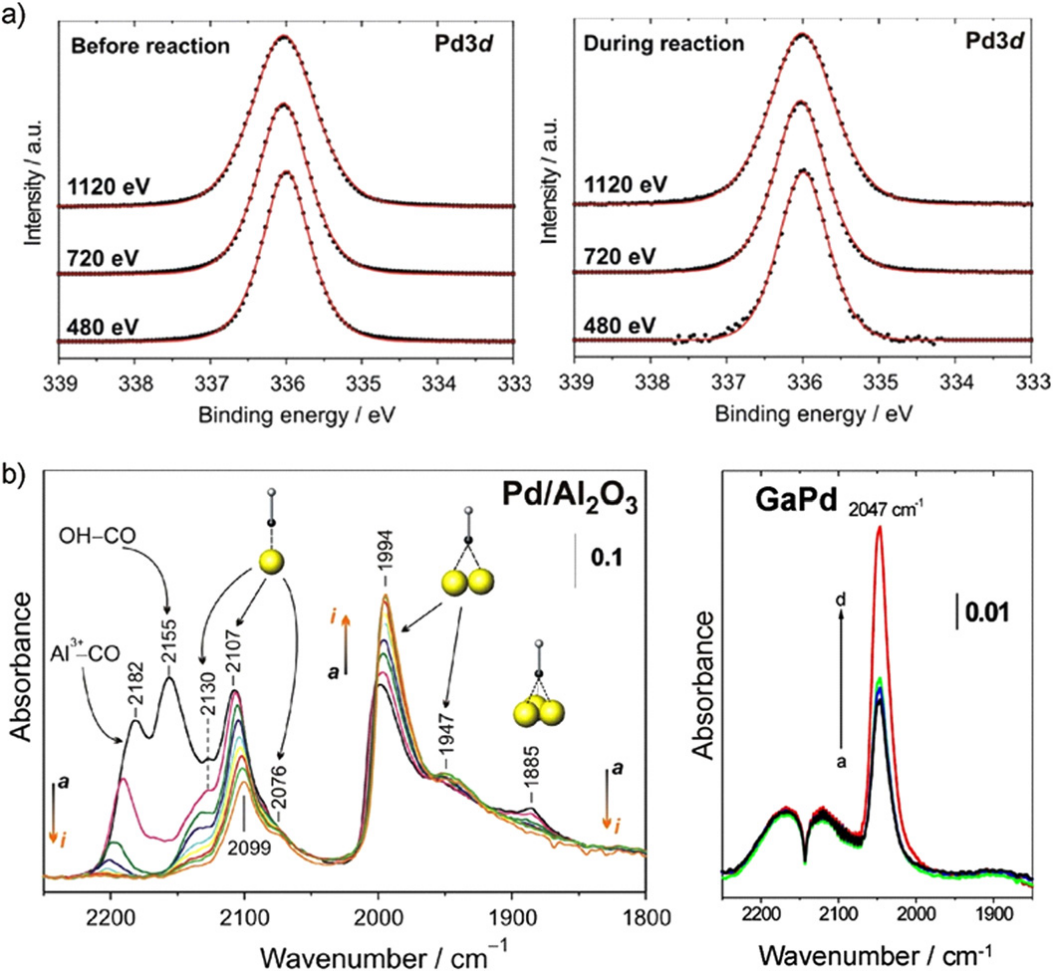
(a) XPS spectra of the Pd 3d_5/2_ region of GaPd in UHV (left) and reactive atmosphere (400 K) (right). (b) Infrared spectra of CO adsorpt on a commercial 5% Pd/Al_2_O_3_ (left, arrows indicate falling partial pressure) and unsupported GaPd powder (right, the arrow indicates increasing partial pressure) revealing only isolated on-top adsorption on GaPd.

The bulk of the compounds thus stays in the state as synthesized, conserving the pre-selected electronic and crystal structures. Even by very sensitive PGAA measurements no hydrogen absorption could be detected [44]. The small amount detected is due to the adsorption of hydrogen and/or hydrocarbons on the surface of the unsupported intermetallic particles. The stability of the bulk is a necessary, but not sufficient, criterion for a successful transfer of the structural and electronic properties of the intermetallic compounds into the reactor. Since the semi-hydrogenation of acetylene takes place on the surface of the compounds, the near-surface region was probed by near-ambient pressure XPS in a 10:1 hydrogen:acetylene mixture (1.1 mbar total pressure, 400 K) [44]. Elemental palladium shows a rich sub-surface chemistry, i.e. incorporation of hydrogen and/or carbon in the first outer atomic layers. This sub-surface chemistry strongly changes the material’s characteristics by influencing the electronic and crystal structure, thereby the hydrogen diffusion properties and thus the catalytic behavior [25]. To detect possibly ongoing sub-surface chemistry as in the case of elemental palladium, depth profiles of the materials were recorded by variation of the incident photon energy (figure 7).

Independent of the information depth—around 4 nm, 2.5 nm and 1.5 nm for 1120 eV, 720 eV and 480 eV, respectively—only one palladium signal is observed as expected from the well-ordered crystal structure under ultrahigh vacuum (UHV) conditions. In a reactive atmosphere, the recorded signals do not change. Comparison of the obtained spectra of palladium and gallium as well as carbon and oxygen under ultra-high vacuum conditions and under *in situ* conditions did not reveal any changes, thus excluding sub-surface chemistry, hydride formation or decomposition of the intermetallic compound. The observed stability is in agreement with the quantum chemical calculations, showing a mixture of ionic and covalent bonding for the Ga–Pd intermetallic compounds. Thus, besides the bulk, also the surface characteristics of the compounds are retained under reaction conditions.

Further information on the state of the palladium atoms on the surface can be gained by employing carbon monoxide as a test molecule and determining its state on the surface by infrared spectroscopy. On Pd/Al_2_O_3_, representing large active sites, CO adsorbs on the support as well as on the palladium surface. On the latter CO adsorbs on top, in bridging and in hollow sites. In addition, a shift of the infrared bands with variation of CO partial pressure is observed. While the first effect results from the different adsorption sites provided by palladium ensembles, the latter results from dipole–dipole coupling [45, 46] between the adsorbed CO molecules and proves their close distance. The recorded spectra of CO adsorbed on GaPd show a very different situation. Only one signal is recorded, being assigned to CO molecules adsorbed on top of the palladium atoms—despite its rather different vibrational frequency. With varying CO partial pressure no change of the vibrational frequency is observed, excluding dipole–dipole coupling between the CO molecules, thus indicating a rather large distance between them [44]. All three effects would be predicted for GaPd: the strong change of the electronic structure alters the adsorption strength of CO, as shown above by TDS, resulting in a change of the on-top vibrational frequency. In addition, CO should only be able to adsorb in the on-top position due to the absence of Pd ensembles and with 3.00 Å the distance between two palladium atoms is too large to allow dipole–dipole coupling between the adsorbed CO molecules.

**Figure 8:**
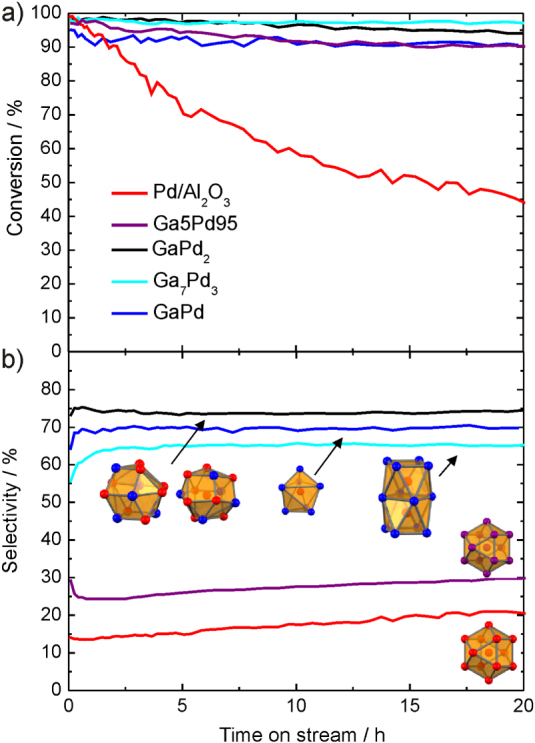
Conversion of acetylene (a) and selectivity to ethylene (b) for the intermetallic compounds Ga_7_Pd_3_, GaPd and GaPd_2_ in comparison to 5% Pd/Al_2_O_3_ and an unsupported Ga5Pd95 alloy (identical color code in both panels).

As for the *in situ* studies, the catalytic properties were determined on crushed single-phase samples (particle diameter of 20–32 *μ*m) to work with as well-defined material as possible [47, 48]. All compounds show activity in the semi-hydrogenation of acetylene in the presence of large amounts of ethylene (figure 8).

Since the conversion has a strong influence on the selectivity (the less strongly adsorbing acetylene is left in the reactant stream, the lower the selectivity) the mass of the materials in the reactor was adjusted to result in 90–95% acetylene conversion. For comparison, commercial 5% Pd/Al_2_O_3_ as well as the unsupported substitutional alloy Ga5Pd95 were tested under identical conditions (473 K, 0.5% C_2_H_2_, 5% H_2_, 50% C_2_H_4_ in helium). Due to the low specific surface areas of the unsupported materials, the specific activity is orders of magnitude lower than for the supported Pd/Al_2_O_3_. But the advantages are clearly seen in the selectivity differences. Using the definition as stated in [48], at a selectivity of 100% acetylene is only converted to ethylene, while at 50% selectivity the acetylene is fully hydrogenated to ethane. Values below 50% express that additional ethane is formed by the unwanted hydrogenation of ethylene. The influence of the isolation of the active sites can directly be seen by going from elemental palladium to the substitutional alloy Ga5Pd95. The selectivity increases from 20 to around 30% due to the better active site isolation. Here, the low gallium content is maybe compensated by enhanced segregation of gallium in this system resulting in a better isolation of the active sites. Turning to the intermetallic compounds an excellent selectivity of around 70% is obtained, showing clearly a different class of materials. Over 20 h nearly no deactivation or loss of selectivity is observed for the intermetallic compounds—in strong contrast to the Pd/Al_2_O_3_. The high selectivity of the Ga–Pd compounds prevents the formation of carbonaceous deposits, which would result in deactivation.

The rather similar selectivities of the different Ga–Pd compounds—with strong differences in their respective electronic structures (cf figure 8)—suggest, under the assumption of their termination in a bulk structure, that the site isolation as a geometric argument is more relevant for binding acetylene than the modification of the local Pd electronic structure in the different crystal structures. This view is corroborated by first quantum chemical calculations of the semi-hydrogenation over intermetallic compounds [49]. These were carried out on the (210) surface, which has so far not been investigated experimentally concerning surface-restructuring and the terminating elemental species. However, combined experimental scanning tunneling microscopy (STM) as well as low-energy electron diffraction recording the voltage dependent intensity (LEED I/V) and quantum chemical density functional theory (DFT) studies of the (111) and (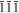) surfaces of GaPd show that these surfaces are not reconstructing, thus exposing the structural arrangement of the atoms as expected from cutting the bulk crystal structure [50]. Comparison of the obtained and calculated LEED data also leads to the conclusion that both surfaces are terminated by palladium atoms—a view not shared by DFT-based calculation of the STM pictures [51]. Further investigations are necessary to clarify this discrepancy.

The concept to use unsupported intermetallic compounds to realize active-site isolation in catalytic materials has proven to be useful to introduce new materials into catalysis. Two immediate question arise: is it possible to transfer the excellent properties of the unsupported materials to high-performance catalysts? And, is a replacement of palladium by a cheaper metal feasible?

## Noble metal-free materials

The quest to replace noble metals or nickel—the latter due to its toxicity—in hydrogenation reactions is long standing. While in heterogeneous catalysis hydrogenation catalysts not containing noble metals require high pressure and temperature, advance has been made in homogeneous hydrogenation, where the first Fe-based materials were identified in the last few years [52, 53]. As mentioned earlier, for the semi-hydrogenation the catalyst should not only be active, but also possess high ethylene selectivity.

**Figure 9:**
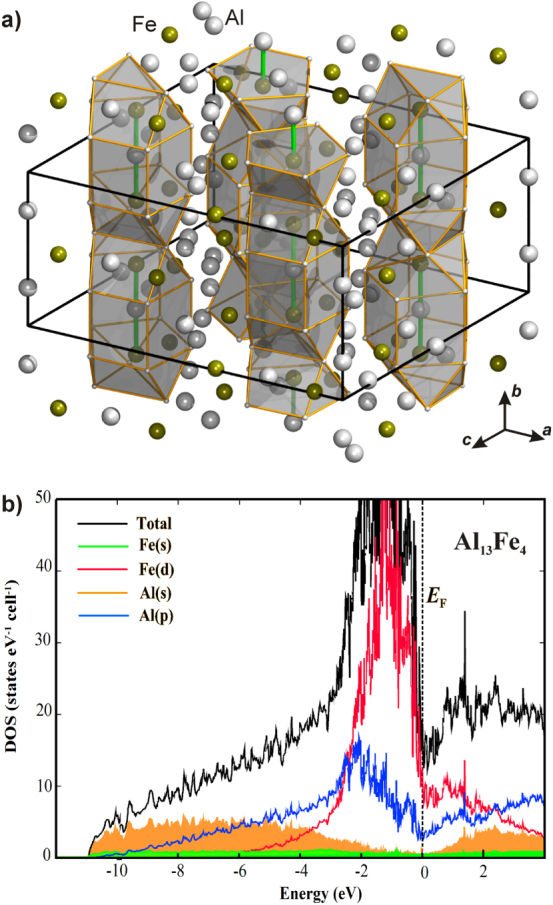
Crystal structure of Al_13_Fe_4_ highlighting the Fe–Al–Fe groups and their surroundings (a). Electronic density of states of Al_13_Fe_4_ (b).

The knowledge-based approach using unsupported intermetallic compounds successfully introduced these materials to the semi-hydrogenation of acetylene and proved the active-site isolation concept. If the conclusions drawn above are correct, electronic effects are playing a minor role. This should allow replacing palladium by another transition metal, which on the one hand provides small and isolated transition metal ensembles. On the other hand, it will not be a disadvantage if the electronic structure resembles the one of the Ga–Pd intermetallic compounds. Besides these requirements from a catalytic point of view, covalent bonding is necessary to stabilize the specific crystal and electronic structure of the intermetallic compound. In addition, the compound has to be resistant against hydride formation to avoid full—and thus unselective—hydrogenation. Based on these considerations, iron was chosen as the transition metal. Due to the expected covalent interactions, aluminum is considered a good partnering metal. In the Al–Fe phase diagram a number of compounds are known, out of which several are line compounds (i.e. are formed at constant composition) [16]. The small homogeneity range is usually a good indicator that electronic factors play an important role in compound formation, e.g. in the form of covalent and/or ionic interactions. Because of the charge transfer from aluminum to iron (*χ*_Al_ = 1.61; *χ*_Fe_ = 1.83), ionic interactions will be present in intermetallic Al–Fe compounds, but the chemical bonding will likely be dominated by covalent interactions as seen by the complex crystal structures formed especially in the Al-rich part of the phase diagram [16]. Out of the remaining candidates, Al_13_Fe_4_—a complex intermetallic compound, possessing more than 100 atoms in the unit cell [54]—shows an interesting local environment of the iron atoms. On the one hand, there are Fe atoms that have only aluminum atoms as closest neighbors, on the other hand, Fe atoms are arranged in Fe–Al–Fe groups which then in turn are encapsulated by aluminum (figure 9).

**Figure 10:**
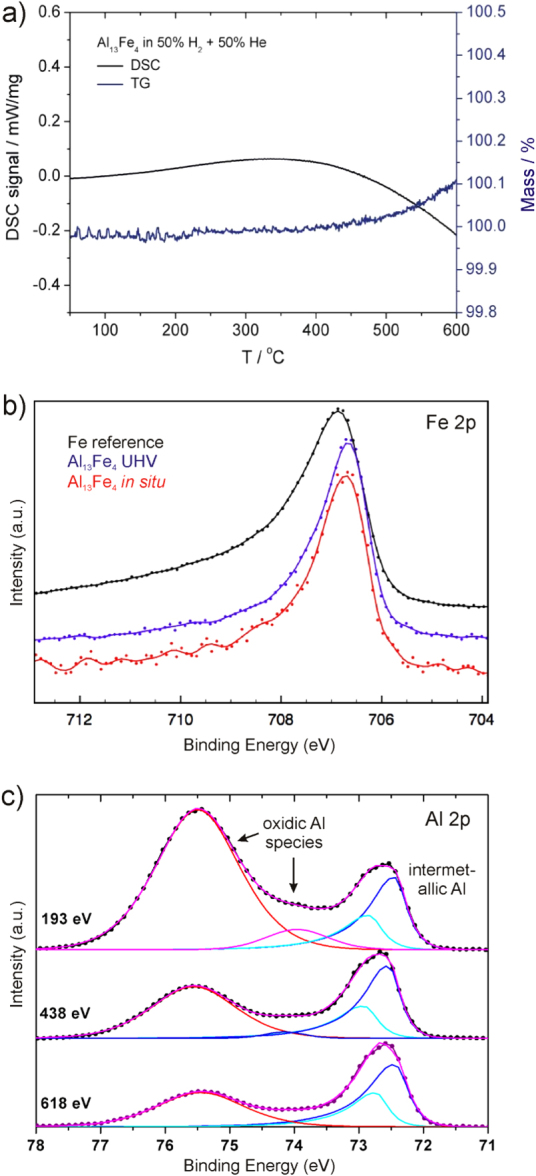
DSC/TG of Al_13_Fe_4_ powder in 50% H_2_/He (a), XPS of the single-crystalline (010) surface: (b) Fe 2p in UHV and *in situ* in comparison to elemental iron foil and (c) depth profile of the Al 2p region corresponding to inelastic mean free paths of 6.6, 11.3 and 14.7 nm (top to bottom).

Calculation of the electronic band structure reveals the expected charge transfer from aluminum to iron and together with the atomic arrangement this leads to a position of the Fe 3d block below the Fermi energy (figure 9(b))—a feature also observed for the Ga–Pd intermetallic compounds (figure 2(b)). Further quantum chemical calculations of the ELI reveal a number of covalent interactions in the compound. This leads to the description of covalently bonded Fe–Al–Fe groups—a feature leading to a clear spatial separation which was verified experimentally by nuclear magnetic resonance and electronic transport property measurements recently [55, 56]. In addition, the presence of iron atoms from the Fe–Al–Fe groups on the [010] surface was recently confirmed under UHV conditions by STM on single crystals [57]. Thus, from an electronic and crystal structure point of view, Al_13_Fe_4_ is a promising candidate. But one further requirement must be fulfilled—the compound must be stable under reaction conditions and not form a hydridic phase. The stability against hydride formation was investigated using bulk sensitive methods and material synthesized by single-crystal Czochralski growth to ensure single-phase samples [58]. Neither PGAA nor XRD measurements in a hydrogen-containing atmosphere showed any changes to the material or a significant hydrogen uptake [59]. DTA/TG measurements in 50% H_2_/He resulted in a high stability up to 400 °C when traces of oxygen start to oxidize the surface of the compound (figure 10). The stability of the near-surface region was further investigated by near-ambient pressure XPS. Comparison of a depth profile before reaction in UHV and under *in situ* conditions (1 mbar hydrogen, 0.1 mbar acetylene) did not reveal any differences, thus excluding changes under reaction conditions. The iron spectra show the presence of a single, clearly altered iron species compared to elemental iron. In addition, as shown by the Al spectra, a thin surface oxide layer is present. Since the signal of Al in the intermetallic compound is clearly visible—even in the most surface sensitive measurements—a closed layer is unlikely, making the intermetallic surface accessible for the reactants.

The material was subsequently tested in an unsupported state in the semi-hydrogenation of acetylene. In contrast to elemental aluminum and iron, the intermetallic compound showed a catalytic activity comparable to the Ga–Pd intermetallic compounds. No deactivation of the material was observed during 20 h time on stream. In addition—and in full agreement with the site isolation concept—the observed selectivity of 81% is as high as for GaPd. Compared to an industrial benchmark system, being optimized for this reaction, the intrinsic selectivity of the material is only 6% lower. By now, Al_13_Fe_4_ has also been shown to be a selective hydrogenation catalyst for butadiene at room temperature under very well-defined UHV conditions, enlarging the substrate portfolio [60]. These results clearly show that the replacement of palladium in hydrogenation reactions is feasible.

## High-performance materials

To answer basic questions and test new approaches, the use of well-defined and reproducibly obtainable unsupported intermetallic compounds with a rather low specific surface area (∼0.1 m^2^ g^−1^) is tolerable. This drastically changes if the aim is to apply the materials industrially.

The raw material costs demand a high atom efficiency of the catalytic materials—especially in the case of noble metal-based systems. High dispersion of the metallic species on a support can result in high atom efficiency. But even for elemental particles, obtaining a good material is not straightforward and reproducible synthesis requires controlling a large number of parameters—especially in large-scale synthesis [10]. The situation becomes much more challenging if the aim is to synthesize a substitutional alloy in a supported state because variation of the chemical composition of the particles has to be excluded to obtain good catalytic performance. A common way of preparation is impregnation of the support materials with salts of the respective elements with subsequent drying and calcination to obtain a material, which can then be stored until use. The material is filled into the reactor and reduced, before switching the reactant stream to the catalyst. Upon reduction, the substitutional alloy forms, providing the aimed-for catalytic properties. Usually these alloys are composed of transition metals that can be reduced by hydrogen or a mixture of hydrogen and carbon monoxide. But what if the reduction potential is not sufficient like in the case of Ga^3+^?

**Figure 11:**
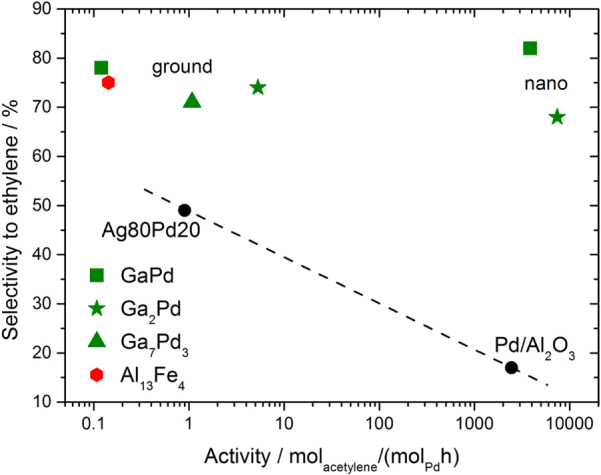
Comparison of several intermetallic catalysts to supported 5% Pd/Al_2_O_3_ and the unsupported substitutional alloy Ag80Pd20. The dashed line is a guide to the eye.

Thus, the challenge one is facing when introducing Ga–Pd intermetallic compounds as catalytic material to industry, is to develop an industrially applicable synthesis to obtain supported intermetallic compounds involving palladium and gallium. Taking on this challenge is worthwhile, as shown by investigating the catalytic properties of nanoparticulate GaPd or GaPd_2_, which have been obtained by a rather expensive and laborious co-reduction in organic solvents making use of a palladium-mediated reduction of the gallium ion [61]. As shown in figure 11, the activity per palladium atom of these materials reaches that of commercially available supported palladium catalysts while preserving the excellent selectivity of the unsupported materials.

One of the challenges becomes obvious when investigating the nanoparticulate materials in detail. A series of scanning transmission electron microscopy investigations on unsupported GaPd_2_ nanoparticles reveals that the short contact to air when preparing the samples is already sufficient to alter their surface [62–64]. Intermetallic gallium in GaPd_2_ seems to be oxidized by air, leaving behind a Pd-enriched near-surface layer. Due to the close relationship of the Co_2_Si type of crystal structure to the cubic closed-packed structure of palladium, the surface of the particles is partially restructuring. The sensitivity of the nanostructured intermetallic compounds to air suggests that the intermetallic nanoparticles are ideally formed in the last step of catalyst preparation, i.e. during reduction.

As result of the promising catalytic properties, a number of promising synthesis routes to supported materials has been developed recently. The simplest route to supported intermetallic nanoparticles is a wet impregnation of carbon nanotubes [65]. While small (2–3 nm) supported intermetallic particles of GaPd_2_ result during the subsequent reduction of the material, the catalytic properties are different from the nanoparticulate ‘benchmark’ synthesized in organic solvents. While the activity is similar to the particles synthesized by co-reduction, the selectivity suffers and drops to below 60%. A size effect can be excluded—otherwise the benchmark with a similar particle size should show the same selectivity. The lower selectivity could result from a support influence, which would rather be unexpected for CNTs or some of the palladium is not fully converted to intermetallic GaPd_2_. Most likely traces of oxygen during the reduction step resulted in the partial decomposition of the intermetallic surface as observed for the unsupported particles after air contact. Another way to prepare supported catalysts is by reactive metal-support interaction. Starting from supported Pd/Ga_2_O_3_, strong reduction in hydrogen above 673 K leads to the formation of Ga–Pd intermetallic compounds [66–68]. The ongoing processes have been explored in detail and depending on which modification of Ga_2_O_3_ is used, it is possible to derive well-defined catalytic materials by this protocol [69]. Interestingly, by palladium-mediated reduction of Ga^3+^ only gallium ions close to the Pd particles are reduced and subsequently diffuse into the palladium particles—as also observed in the organic solvent route to unsupported nanoparticles. In consequence, the palladium loses its ability to form hydrides—being responsible for the activation of hydrogen—and the reduction of Ga_2_O_3_ stops. Consequently, this self-limiting formation mechanism results in a rather homogeneous material. For the simultaneous synthesis of supported intermetallic nanoparticles and the support an industrially applicable, scalable and water-based synthesis protocol using cheap starting materials has been developed by Behrens *et al* [70, 71]. Starting from well-defined hydrotalcites as precursor materials, the intermetallic compounds are formed in a nanoparticulate state during careful reduction. Interestingly, these materials show a long activation period in the semi-hydrogenation of acetylene, resulting in very active (28 600 mol_acetylene_/mol_Pd_h) and selective materials (70% at 97% conversion). The ongoing processes during activation are not understood yet but seem to involve a restructuring of the nanoparticles.

However, the nanostructured materials may exhibit different properties than the bulk materials due to changes on their surface. Exposure to air can alter the surface as shown above, but this can also happen under reductive conditions if very small amounts of water are present. The liquid-phase hydrogenation of phenyl acetylene over bulk and nanoparticulate GaPd_2_ is the first example, where the changes of the surface have been monitored closely [72, 73]. Traces of water being present in the solvents result in an oxidic overlayer in which palladium is embedded, yielding a supported catalyst best described as Pd/Ga_2_O_3_/GaPd_2_. Since the reactants come in contact with the very reactive elemental palladium first, low selectivity to the semi-hydrogenation product styrene is observed. Experimental verification of these processes was given by a thorough surface characterization of the materials in various states of exposure with and without oxygen species. This view is further corroborated by the observation that during extremely dry hydrogenation conditions the expected high selectivity to styrene is observed.

In conclusion, the excellent catalytic properties of unsupported intermetallic bulk compounds can be transferred to high-performance materials if the synthetic hurdles can be overcome. A change of reaction conditions—even if this is restricted to going from the gas to the liquid phase—can result in an altered surface of the compounds, resulting in different catalytic properties. These observations bring up a new interesting question: how widely are intermetallic compounds applicable in heterogeneous catalyzed processes? How severely oxidizing can the reaction conditions be? A first step towards a systematic exploration of the potential of intermetallic compounds in catalysis has been taken in the last few years by exploring intermetallic compounds as catalysts for methanol steam reforming (MSR).

## Methanol steam reforming

MSR is not only an interesting reaction to test intermetallic compounds concerning their stability in stronger oxidizing atmospheres, but also most likely an important building block of our future hydrogen-based energy infrastructure [8, 74]. During MSR, methanol and water react to hydrogen and carbon monoxide in a 3:1 ratio (reaction (1)). Besides the steam reforming also the decomposition of methanol, leading to hydrogen and carbon monoxide can occur (reaction (2)). The water gas shift reaction connects the products CO, CO_2_ and hydrogen as well as the reactant water (reaction (3)).

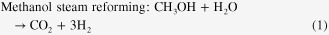


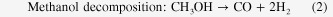


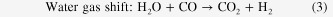



Iwasa and co-workers introduced supported intermetallic compounds as catalysts in this reaction in the 1990s [75, 76]. Looking for a catalytic system that can circumvent the deactivation and pyrophoric behavior of the Cu/ZnO/Al_2_O_3_ catalysts (originally developed for the inverse reaction, i.e. methanol synthesis), Iwasa tested palladium and platinum particles supported on different oxides. Applying a hard-to-reduce oxide, e.g. SiO_2_, resulted in methanol decomposition into carbon monoxide and hydrogen as it is expected for elemental palladium. In strong contrast, a very high selectivity toward MSR resulted if the supporting oxide was rather easy to reduce like ZnO. Characterization of the materials after the catalytic tests revealed the formation of intermetallic compounds, e.g. ZnPd, if an easy-to-reduce oxide like ZnO was used as the supporting material. This observation suggested that ZnPd seems to be stable under MSR conditions and possesses an improved long-term stability compared to the Cu-based catalysts.

The promising catalytic properties triggered great interest in the behavior of different intermetallic compounds in this reaction. Here, the complexity of the supported materials complicates gathering the sought-for deep understanding of the role of the different components. In consequence unsupported intermetallic compounds were tested as catalysts in this reaction [77]. Focusing on structurally similar compounds, geometric differences were minimized and the influence of the different electronic structures on the catalytic properties was explored. Catalytic tests on ZnPd, ZnPt, ZnNi and CdPd in comparison to elemental palladium and copper resulted in a correlation of the CO_2_ selectivity with the electronic structures of the compounds. ZnPd and CdPd exhibit a valence band structure which is similar to copper, i.e. the electronic DOS at the Fermi energy is low and the upper energy of the d-band lies around −1.5 eV, resulting in a very high CO_2_ selectivity (∼95%) during MSR. On the other hand, the d-band of ZnNi, ZnPt and elemental palladium lies closer to or on the Fermi energy, resulting in a high DOS at low binding energy and thus in a strong dehydrogenation activity resulting in much CO and thus a lower CO_2_ selectivity (ZnPt 40%; ZnNi 7%; Pd 1%) [77, 78]. While this is a relevant conceptual observation, the *in situ* stability of the compounds—which can have a crucial influence as seen above—was not under investigation in these early studies.

Later work on unsupported ZnNi revealed that the compound is decomposing under reaction conditions, thus the material under reaction conditions is not the intermetallic compound anymore, but a mixture of oxidized species [79]. As result, the electronic structure under reaction conditions is no longer that of the intermetallic compound, but that of the decomposition products. Thus, a correlation between the observed catalytic properties and the electronic and/or crystal structure of the intermetallic compound is not meaningful under these circumstances.

**Figure 12:**
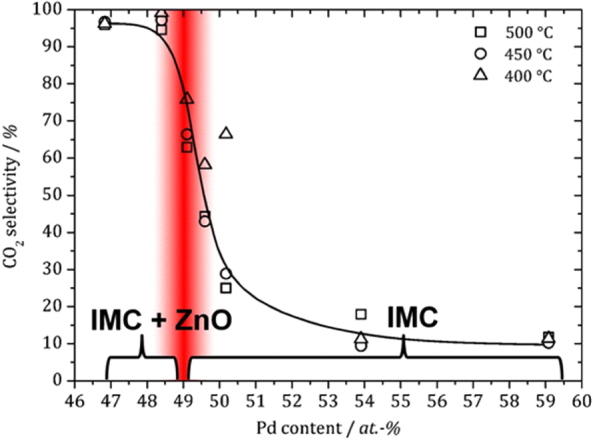
Composition dependent CO_2_ selectivity in MSR at different temperatures over unsupported ZnPd. Results from *in situ* ambient pressure XPS measurements are summarized at the bottom (IMC: intermetallic compound), revealing the presence of ZnO in the case of samples rich in Zn.

Besides ZnNi, also the compound ZnPd was investigated with regard to its stability under reaction conditions. In addition, ZnPd possesses a broad homogeneity range of more than 10 at. %. Deviations from the ideal composition result in changes in the electronic structure—due to the varying number of electrons per unit cell—as well as structural alterations (vacancies, anti-site occupancy or interstitial sites) caused by accommodation of additional Zn or Pd atoms in the unit cell. Thus, a composition-dependence of the catalytic properties is expected. A close look on ZnPd under reaction conditions reveals a composition-dependent partial oxidation of the near-surface region [80]. The broad homogeneity range allows for the synthesis of unsupported Zn-rich and Pd-rich samples besides material with equimolar composition. Under reaction conditions, the Zn-rich samples oxidize partially in such a way that some intermetallic surface is still accessible. Pd-rich samples on the other hand do not oxidize. As a result, ZnO and ZnPd are present in the Zn-rich samples under reactions conditions, while in Pd-rich samples only the intermetallic surface is exposed. This has strong implications on the catalytic properties of the samples: whenever the intermetallic compound and the oxide are present, excellent activity and selectivity to CO_2_ (up to 99.6%) results. In contrast, Pd-rich samples without ZnO being present show low activity and very low CO_2_ selectivity (∼10%) (figure 12).

These changes to the intermetallic surface are not restricted to unsupported and single-phase materials, but also occur on the originally introduced material ZnPd/ZnO. After the last step of preparation, i.e. directly after strong reduction at 773 K in 10% H_2_, ZnPd nanoparticles are observed by TEM [81]. These particles are not single crystalline, but composed of a number of ZnPd crystallites and do not show any deviation from the clean intermetallic surface. Introducing this material into a reactive atmosphere results in a very low CO_2_ selectivity of only 40% in the beginning—despite the fact that ZnPd as well as ZnO are present. In the first hour of the catalytic experiment the selectivity to CO_2_ is strongly increasing, reaching >97% after 3 h on stream. TEM investigations of the material in its highly selective state reveal the formation of small ZnO islands decorating the now much better ordered ZnPd particles. Thus, switching from strongly reducing to reaction conditions leads to partial oxidation of the intermetallic surface, leading to a strong increase in the abundance of the ZnPd–ZnO interface, which in turn results in high CO_2_ selectivity. From these observations, it seems that the intermetallic compound ZnPd is able to activate only one of the components, i.e. methanol. Thus, if only ZnPd is present as in the case of the Pd-rich unsupported samples only methanol decomposition is taking place, resulting in very low CO_2_ selectivity.

**Figure 13:**
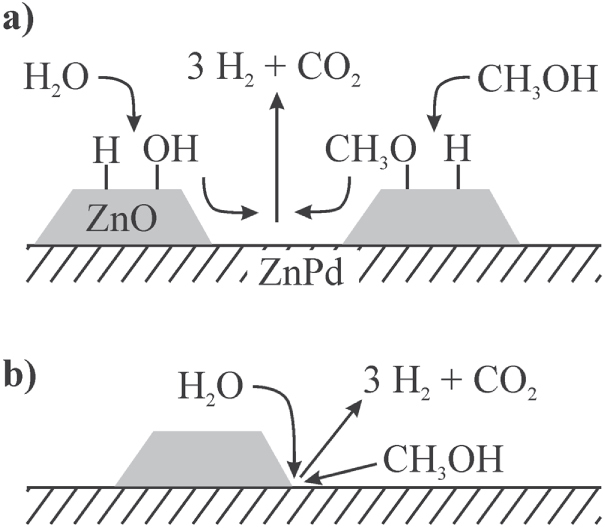
Two pathways for methanol steam reforming: the ZnPd/ZnO interface holds the active sites (a) or the reaction proceeds via spill-over of activated species from ZnPd and/or ZnO (b).

To activate the other reagent, i.e. water that leads to oxidation of CO, ZnO must be present—which in its own right is a very selective MSR catalyst with low activity [82]. In the case of the unsupported Zn-rich ZnPd, ZnO is formed by oxidation of the intermetallic compound leading to the excellent CO_2_ selectivity. On the supported material, the gain in abundance of the ZnPd–ZnO interface within the first hours leads to increasing selectivity. In one possible scenario—explaining all experimental observations—ZnO activates the O–H bonds in both molecules, while ZnPd is responsible for the C-H activation. From this, two possible ways for the reaction to proceed can be developed: either the ZnPd–ZnO interface is holding the active sites, or a spill-over from O-H activated species from ZnO to ZnPd and/or a spillover from activated methanol from ZnPd to ZnO takes place (figure 13).

The investigations above show the alterations of intermetallic compounds like ZnPd and ZnNi under reaction conditions, making a correlation of their electronic and crystallographic structure to the catalytic properties not as straightforward as in the case of the semi-hydrogenation in the gas phase. In the case of ZnPd, even exposure to carbon monoxide results in structural modifications [83, 84], preventing the use of this frequently used test molecule in heterogeneous catalysis to determine the nature of the surface of ZnPd-based catalysts. Up to now, the behavior of ZnPd under reaction conditions is not at all explored in a systematic way (for a comprehensive review on ZnPd see [85]), but potentially holds the key for a full understanding of the requirements for MSR catalysts and, thus, the development of innovative materials combining higher activity and selectivity.

The changes of the surface have also to be considered when comparing experimental results to the numerous quantum chemical calculations on different ZnPd and ZnO surfaces (see e.g. [86, 87] as well as [85] for a recent review). The complexity of the ZnPd–ZnO interface as well as the presence of methanol, water and the different reaction products has so far hindered a quantum chemical calculation taking possible beneficial effects of the interface into account. Nevertheless, quantum chemistry would be a great help to differentiate between the two reaction paths mentioned above.

The change of intermetallic compounds under reaction conditions is most unfortunate if one aims at setting up structure-property relationships based on the electronic and crystallographic structure of the intermetallic compound. But this disadvantage can also be turned into a potential to synthesize materials with new properties by deliberate decomposition of the intermetallic compounds, thus making the decomposition part of an alternative synthesis route. The intermetallic compound is then used as a precursor with a controllable residual chemical reactivity turning upon suitable activation into a nanostructure with homogeneous properties as their constituting atoms come from a homogeneous and well-defined parent structure with atomic dispersion. This approach was first introduced into catalysis by Raney [88, 89] with the selective leaching of Ni–Al and Ni–Si alloys (i.e. mixtures of different intermetallic compounds and substitutional alloys) with sodium hydroxide solution to obtain highly active Ni catalysts. The same concept of a homogeneous intermetallic compound as precursor for relevant catalysts was developed with self-supporting amorphous intermetallic compounds transformed *in situ* into nanocrystalline highly stable supported systems of uniform size and thus superior catalytic properties. The concept [90–95] developed for ammonia synthesis and CO oxidation was successfully transferred [96] to technical applications in selective hydrogenation using PdSi_*x*_ intermetallic compounds.

**Figure 14:**
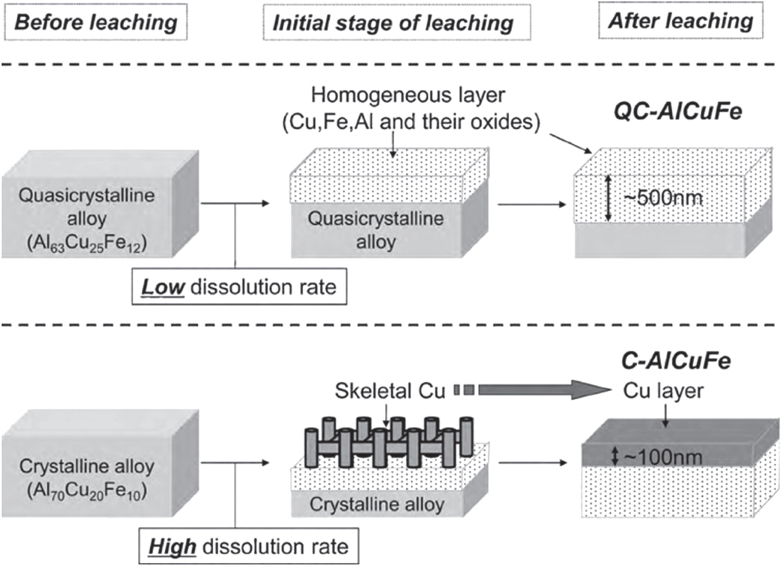
Different leaching behaviors of the quasicrystalline compound Al_63_Cu_25_Fe_12_ and the crystalline compound Al_70_Cu_20_Fe_10_, leading to different Cu morphologies after leaching (adapted with permission from [100]).

Applying the approach to single-phase quasicrystalline Al_63_Cu_25_Fe_12_ resulted in small copper particles on the surface of the intermetallic compound. Subsequent testing of the material in MSR resulted in an activity of 240 mL H_2_ per gram of catalyst per minute at 573 K, very similar to a commercial catalyst tested under identical conditions [97]. A subsequent study [98] revealed the high stability of the copper particles against sintering, overcoming a widespread problem for conventionally synthesized catalysts in this reaction. The stability of the unconventionally synthesized copper particles was assigned to the presence of iron—which is immiscible with copper—as well as to the special interaction of the copper particles with the quasicrystalline surface. Optimizing the milling (in ethanol) and leaching procedure (323 K, aqueous Na_2_CO_3_) the activity could be nearly tripled to 677 mL H_2_/g_catalyst_min at 573 K [99]. Further investigations on the leaching process uncovered a completely different leaching behavior of the quasicrystalline material (Al_63_Cu_25_Fe_12_) in comparison to a conventionally crystallized alloy with similar composition (Al_70_Cu_20_Fe_10_) [100]. The much lower Al-dissolution rate of the first results in the formation of small copper particles, while the crystalline alloy—despite its very similar composition—possesses a higher dissolution rate for Al leading to skeletal copper on the surface (figure 14).

The different chemical behavior of the crystalline intermetallic compound in comparison to the quasicrystalline compound leads to very different materials after leaching. While the quasicrystalline material reveals much higher stability against sintering due to the higher dispersion of the copper particles, the conventional Raney catalyst does not show this beneficial behavior.

## Conclusions

In this short review, the contribution of intermetallic compounds to a knowledge-derived approach in heterogeneous catalysis is highlighted. Intermetallic compounds allow geometric and electronic influences to be addressed, if their stability under reaction conditions is explored. In the semi-hydrogenation of acetylene research on intermetallic compounds has allowed identification of innovative and noble metal-free catalysts, i.e. Al_14_Fe_4_ and Al_13_Co_4_. In addition, the catalytic properties of the unsupported Ga–Pd model catalysts could be transferred to high-performance materials by several routes, including industrially applicable and scalable synthesis protocols.

Exploring the catalytic properties and the *in situ* stability of several intermetallic compounds in MSR revealed a strong synergy between ZnPd and ZnO, leading to a deep insight into this material/reaction combination. The resulting precursor concept is a vivid illustration for the decisive function of chemical dynamics of catalytic materials bringing about their catalytic function only in contact between their precursors and the reactants. Applying and optimizing preferential leaching to quasicrystalline intermetallic compounds resulted in Cu-based materials with superior MSR properties due to the very different chemical behavior of the quasicrystalline materials in comparison to crystalline samples.

It is likely but not yet clearly recognized that the relevant concept of activating Pt nanoparticles for fuel cell applications by *in situ* electrochemical de-alloying [101–103] leads at the surface of the nanoparticles to residual intermetallic compounds explaining the stability of the beneficial reaction properties as compared to monometallic Pt of the same particle size.

Intermetallic compounds have the potential to narrow the materials gap in heterogeneous catalysis due to their availability as nanoparticles, crushed powders and large single crystals. While the first two allow reactor studies, the latter open a large field of surface science investigations. The results on the surface structure of these investigations form the basis for quantum chemical calculations, which deepen understanding of the ongoing processes.

With their huge structural and electronic variety, intermetallic compounds possess a huge potential in heterogeneous catalysis and allow for a systematic investigation of catalytic phenomena. However, their *in situ* stability is a crucial prerequisite for such investigations.
